# PARP-1 inhibitor modulate β-catenin signaling to enhance cisplatin sensitivity in cancer cervix

**DOI:** 10.18632/oncotarget.27008

**Published:** 2019-07-02

**Authors:** Minakshi Mann, Sachin Kumar, Ashok Sharma, Shyam S. Chauhan, Neerja Bhatla, Sunesh Kumar, Sameer Bakhshi, Ritu Gupta, Lalit Kumar

**Affiliations:** ^1^ Department of Medical Oncology, Dr. B.R.A. Institute Rotary Cancer Hospital, All India Institute of Medical Sciences, New Delhi, India; ^2^ Department of Biochemistry, All India Institute of Medical Sciences, New Delhi, India; ^3^ Department of Obstetrics and Gynecology, All India Institute of Medical Sciences, New Delhi, India; ^4^ Laboratory Oncology Unit, Dr. B.R.A. Institute Rotary Cancer Hospital, All India Institute of Medical Sciences, New Delhi, India

**Keywords:** cervical cancer, cisplatin, PARP-1, PJ34, β-catenin

## Abstract

Cisplatin is a keystone for treatment of both recurring and locally advanced cervical cancer. However toxic side effects and acquired resistance limits its efficacy. Enhanced DNA repair is one of the mechanisms through which cancer cells acquire cisplatin resistance. Inhibitors of PARP, which is a DNA damage repair enzyme, have been approved for use in BRCA mutated cancers like breast and ovary cancer. However little is known about the therapeutic efficacy of PARP inhibitors in cervical cancer, either as a single agent or in combination with cisplatin. We hypothesized that PARP-1 inhibition might improve the sensitivity of cervical cancer cells to cisplatin by diminishing DNA repair. To ascertain this, we determined effect of PARP-1 inhibition on cisplatin cytotoxicity in HeLa and SiHa cell lines. Combination of cisplatin with PJ34, a phenanthridinone-derived PARP-1 inhibitor, augmented cisplatin toxicity *in vitro* by decreasing cell proliferation, enhancing cell cycle block and cell death, and decreasing invasion and metastasis, when compared with either of the single agent alone. We further show that PARP-1 inhibition inhibited β-catenin signaling and its downstream components such as c-Myc, cyclin D1 and MMPs indicating a possible link between single strand base damage repair and WNT signaling. In conclusion, PARP-1 inhibition might augment cisplatin cytotoxicity in cervical cancer cells by modulating β-catenin signaling pathway. Combining PARP-1 inhibitors with cisplatin might be a promising approach to overcome cisplatin resistance and to achieve a better therapeutic effect.

## INTRODUCTION

Cervical cancer is a common gynecological cancer among females worldwide with an annual incidence and mortality of 0.53 million and 0.25 million, respectively [1]. In developed countries, a sharp decline in incidence as well as mortality due to this cancer has been observed. However, in developing countries like India, it remains second commonest cancer accounting to 22.8% of total cancer cases among females (http://screening.iarc.fr/doc/WHO_India_CCSP_guidelines_2005.pdf) and contribute to approximately 10% of all cancer related mortality, making it the third leading cause of death [2]. Human papillomavirus 16 (HPV16) and HPV18 belong to high risk group of human papillomavirus and are responsible for more than 80% of all cervical cancer cases [3]. Among various histological subtypes, squamous carcinoma and adenocarcinoma of cervix are major reported sub types [4].

Cisplatin (CDDP), a first generation platinum analogue, is a keystone for the treatment of both recurring and locally advanced cervical cancer [5]. CDDP mediates its cytotoxic effect by causing CDDP-DNA adduct formation thereby leading to apoptosis [6]. Response rate with CDDP-based therapies ranges from 20% to 50% with an expected overall survival remaining low i.e. ranging from 10 months to 17.5 months [7]. Moreover, toxic side effects and acquired resistance are limitations of CDDP-based chemotherapy [8]. Mechanisms implicated in tumors resistance to CDDP include, i) drug inactivation by enhanced drug detoxification through glutathione, ii) increased efflux, iii) inhibition of apoptosis through overexpression of Bcl-2 or inhibition of caspase activity, and iv) increased DNA damage tolerance and repair [6], [9]. Of these, increased DNA damage response might play an important role since several studies have shown that CDDP resistant cells display an increased capacity to repair CDDP-DNA adducts [10]. Hence, in the absence of any other effective chemotherapeutic agent for the management of cervical cancer, improving the efficacy of CDDP remains the major challenge. Therefore, identification of key signaling pathways and genes involved in CDDP resistance is of utmost importance.

Poly (ADP-ribosyl) polymerase (PARP-1) is a nuclear DNA binding protein and a key component involved in single strand break (SSB) damage repair. PARP-1 is the most abundant protein of PARP family [11]. Upon DNA damage, PARP-1 catalyzes the PARylation of acceptor proteins and repairs damaged DNA through base excision repair (BER), thereby maintaining genomic stability [12]. Moreover, recently Michels *et al* demonstrated that cancer cells often develop CDDP resistance due to PARP hyperactivation [13–15]. Use of PARP-1 inhibitors in breast cancer 1 (BRCA1) or breast cancer 2 (BRCA2) mutated tumors leads to synthetic lethality by making them highly sensitive to CDDP and other DNA damaging agents [16, 17]. Therefore, PARP-1 inhibitors (PARPi), either as single agent or in combination with other chemotherapeutic agents, are being extensively explored in tumors bearing defects in homologous recombination (HR) pathways such as breast and ovarian cancer [18, 19]. Numerous phase I and II clinical trials have shown that PARPi olaparib (Astrazeneca/KuDOS) exhibit anti-neoplastic response in patients with BRCA1/2 mutated tumors and reduces risk of recurrence when used as a maintenance therapy [20]. However, there is limited evidence on the combinatorial effect of PARPi with cytotoxic drugs in HPV-associated cervical cancer. Further, the exact effect of PARPi on CDDP sensitivity in cervix cancer and the mechanism of action are poorly understood.

In this study, we have investigated the combined effect of PARP-1 inhibition and CDDP on cell proliferation, survival, apoptosis, and invasion and migration in cervical cancer. Pharmacological (PJ34) and genetic (siRNA) abrogation was used for PARP-1 inhibition. PJ34 ([*N*-(6-oxo-5,6-dihydrophenanthridin-2-yl)-*N*, *N*-dimethylacetamide. HCl]), is a water-soluble phenanthridinone-derived PARP-1 inhibitor that acts as a competitive inhibitor of NAD binding to PARP-1 active site; thereby inhibiting its enzymatic activity [21]. Our findings indicate that PARP-1 inhibitor potentiates the antineoplastic effects of CDDP. Interestingly, we observed that PARP-1 inhibitor enhances anti-neoplastic effect of CDDP by inhibiting the expression of beta-catenin (ß-catenin) and its down-stream signaling components like MMP, c-Myc and cyclin D1. This is the first report, which shows that PARP-1 inhibition enhances CDDP sensitivity by targeting ß-catenin signaling.

## RESULTS

### PJ34 inhibits the growth of cervical cancer cells, but less effectively than CDDP

**Figure 1:**
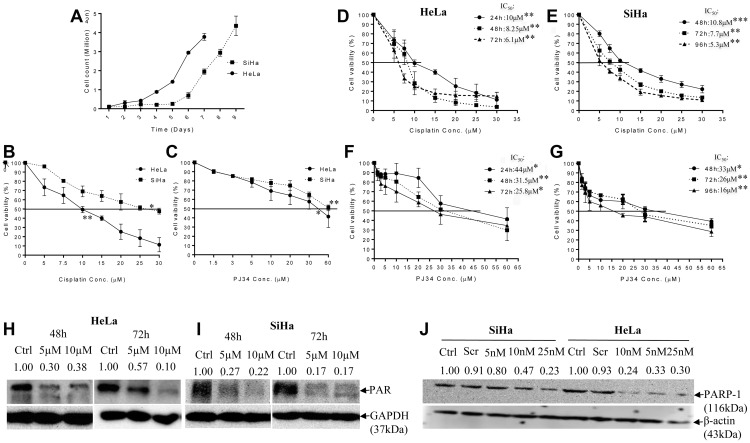
Doubling time and dose-response effect of PJ34 & CDDP on cell vaibility and representative immunoblots confirming PARP-1 inhibition. (**A**), cell doubling time of the HeLa and SiHa cells as a function of the initial seeding density. The cell doubling time of HeLa and SiHa cells is 22.06 h and 40.33 h respectively. Doubling time was calculated through website: http://www.doubling-time.com/compute.php, using formula: DoublingTime = duration-log(2)log/log(FinalConcentration)−log(InitalConcentration). Where “log” is the logarithm to base 10 or 2 or any other base. (**B** and **C**), HeLa and SiHa cells were treated with indicated doses of CDDP and PJ34 for 24 h and cell viability was determined by MTT assay. All the values are expressed relative to untreated cells (100% control value). (**D**–**G**), cells were treated with indicated doses of PJ34 & CDDP for 24–72 h (HeLa) & 48–96 h (SiHa) and cell viability was determined by MTT assay. All the values are expressed relative to untreated cells (100% control value). (**H–J**), immunoblots showing endogenous level of PAR in HeLa and SiHa cells after treatment with PJ34 (0, 5 and 10 μM) for 48 h–72 h and PARP-1 expression after treatment with PARP-1 siRNA (0, 5, 10 and 25 nM) in HeLa and SiHa. GAPDH and β-actin was used as loading control. Error bars represent mean ± SD (*n* ≥ 3 independent experiments). IC_50_ values for CDDP and PJ34 at different time points along with their p value is mentioned in the respective graph. ^*^ *p < 0.05;* ^**^ *p<0.01;* ^***^ *p<0.001.*

We determined and compared the cytotoxic efficacy of PJ34 and CDDP as single agents. Cell survival in presence of increasing concentration of PJ34 and CDDP was assessed by MTT assay at three different time points depending upon the doubling time of respective cell line (HeLa: 22.06 h & SiHa: 40.33 h; Figure 1A). A dose-dependent decrease in cell survival was observed and the IC_50_ values for CDDP were evaluated to be 10 μM (24 h), 8.25 μM (48 h) and 6.1 μM (72 h) for HeLa cells (Figure 1D) and 10.8 μM (48 h), 7.7 μM (72 h) and 5.3 μM (96 h) for SiHa cells (Figure 1E). The corresponding IC_50_ values for PJ34 were 44 μM (24 h), 31.5 μM (48 h) and 25.8 μM (72 h) for HeLa cells (Figure 1F) and 33 μM (48 h), 26 μM (72 h) and 16 μM (96 h) for SiHa cells (Figure 1G). These results indicate that CDDP is more cytotoxic and SiHa cells were comparatively more resistant to both the drugs than HeLa cells (Figure 1B and 1C).

### PARP-1 abrogation enhances CDDP sensitivity in cervical cancer cells

**Figure 2:**
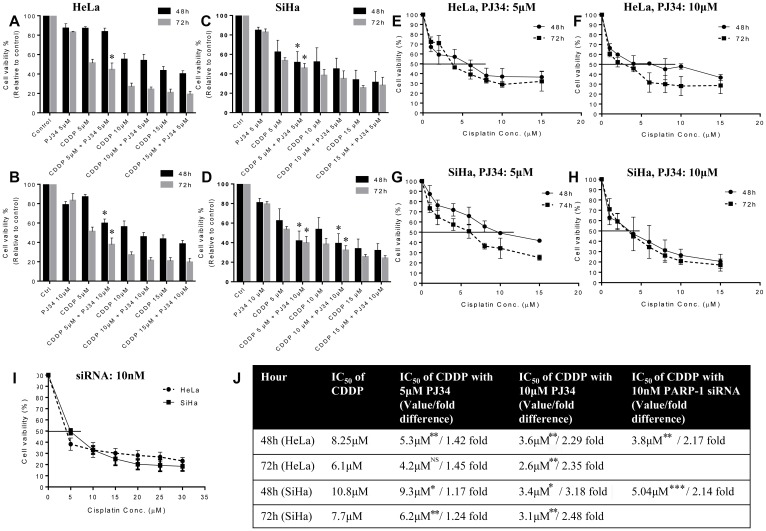
Dose-response effect of CDDP (5, 10 and 15 μM) in combination with PJ34 (5 and 10 μM) on cell vaibility and IC_50_ value of CDDP at different time points. (**A–D**), HeLa cells were treated with different doses of CDDP in combination with 5 μM (A) or 10 μM (B) of PJ34. SiHa cells were treated with different doses of CDDP in combination with 5 μM (C) or 10 μM (D) of PJ34. (**E–I**), HeLa cells were treated with different doses of CDDP in combination with 5 μM (E) or 10 μM (F) of PJ34. SiHa cells were treated with different doses of CDDP in combination with 5 μM (G) or 10 μM (H) of PJ34. HeLa and SiHa cells were treated with 10 nM of PARP-1 siRNA in combination with various doses of CDDP (I). Table representing fold change in the IC_50_ value of CDDP upon combined treatment with PARP-1 inhibitor and CDDP vs. CDDP alone in HeLa and SiHa cells (**J**). Error bars represent mean ± SD (*n* ≥ 3 independent experiments). IC_50_ values for combined treatment with PJ34 and CDDP at different time points along with their p value is mentioned in the table. ^*^ *p < 0.05;* ^**^ *p<0.01;* ^***^ *p<0.001.*

As aforementioned in methodology, for CDDP 5 μM, 10 μM and 15 μM was chosen as optimal concentration for evaluating the effect of PARP inhibition on CDDP sensitivity. A concentration of 5 μM and 10 μM of PJ34 was found to significantly inhibit PARP-1 activity (PARylation) in both the cell lines as shown by immuno-blots (Figure 1H and 1I). Genetic abrogation of PARP-1 by siRNA (at 10 nM concentration) was also able to efficiently suppress PARP-1 expression (Figure 1J). We didn’t observe any significant drug-related cytotoxicity in the culture at above-mentioned concentrations of PJ34 and siRNA. In combinatorial treatment, a significant decrease in cell survival was observed when PJ34 was combined with CDDP at various time points in HeLa (Figure 2A and 2B) and SiHa cells (Figure 2C and 2D) and this combination of CDDP and PJ34 was found to act in synergistic manner (Supplementary Table 1). Combining PJ34 with CDDP also significantly reduced the dose of CDDP required to achieve 50% cytotoxicity (IC_50_ values) in both the cell lines (Figure 2E–2H, 2J). With 10 μM PJ34, the IC_50_ value of CDDP decreased 2.29 fold and 2.35 fold at 48 h and 72 h, respectively in HeLa cells (Figure 2F). In SiHa, the IC_50_ value of CDDP decreased 3.18 fold and 2.48 fold at 48 h and 72 h, respectively (Figure 2H). Similarly, silencing of PARP-1 by 10 nM siRNA also significantly reduced IC_50_ of CDDP by 2.17 fold in HeLa cells and 2.14 fold in SiHa cells at 48 h (Figure 2I). These results confirm that pharmacological or genetic abrogation of PARP-1 enhances the CDDP cytotoxicity in cervical cancer cells.

### PARP-1 abrogation enhances CDDP-mediated cell cycle block and cell death in cervical cancer cells

**Figure 3:**
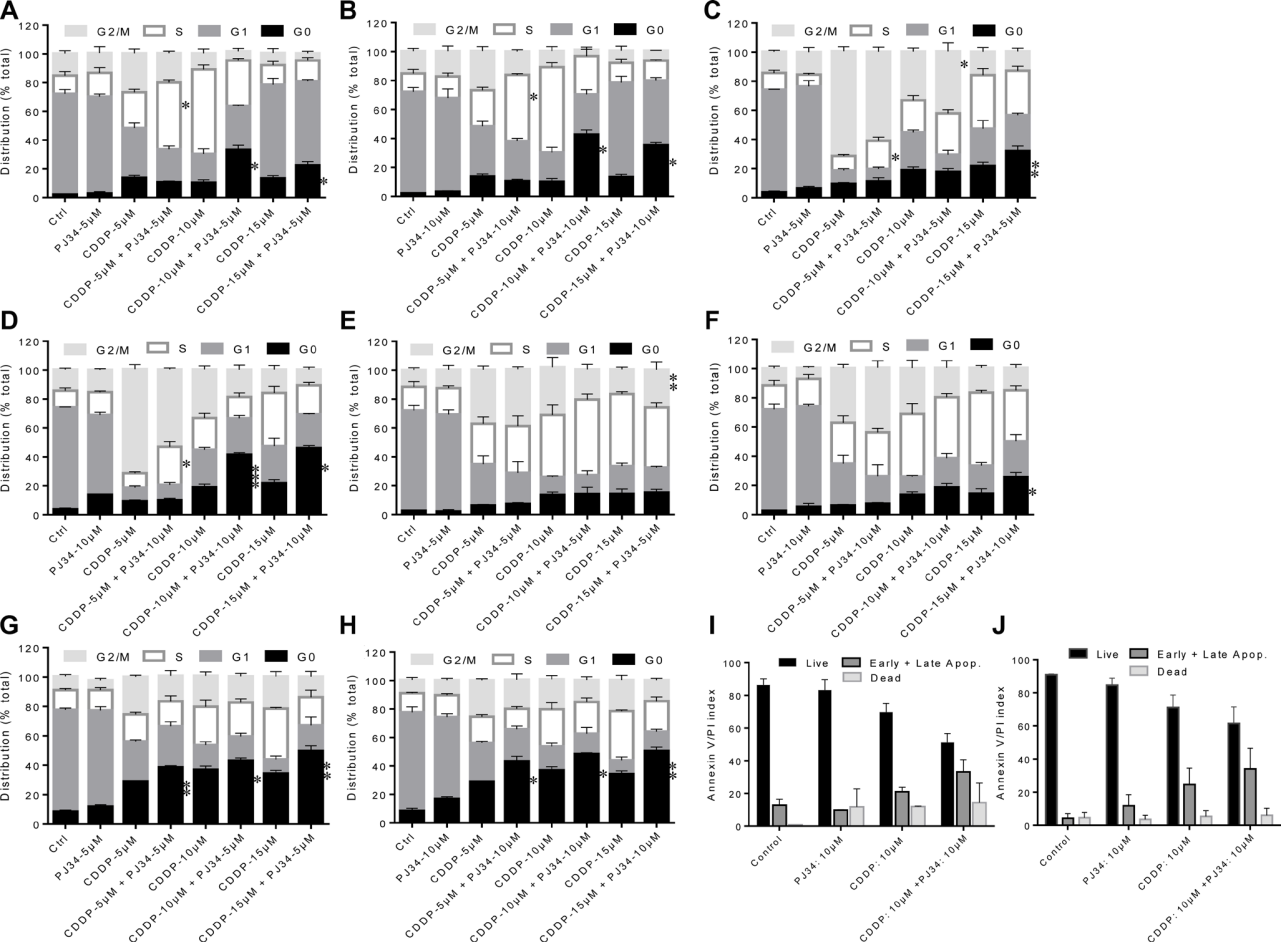
Combined effect of PJ34 & CDDP on cell cycle progression and apoptosis. (**A–H**) HeLa cells were treated with different doses of CDDP in combination with 5 μM of PJ34 and 10 μM of PJ34 for 48 h (A and B) and 72 h (C and D). SiHa cells were treated with different doses of CDDP in combination with 5 μM of PJ34 and 10 μM of PJ34 for 72 h (E and F) and 120 h (G and H). (**I** and **J**), cells were treated with indicated doses of CDDP in combination with 10 μM of PJ34 for 48 h (HeLa, I) and 72 h (SiHa, J). Error bars represent mean ± SD (*n* ≥ 3 independent experiments). ^*^ *p < 0.05;* ^**^ *p < 0.01;* ^***^ *p < 0.001.*

We next analyzed the effect of PJ34 and CDDP treatments alone or in combination on the cell cycle and apoptosis. CDDP has been known to increase the duration of S-phase and blocks cells in G2 phase in a dose- and time-dependent manner followed by cell death [22, 23]. CDDP is not cell cycle specific and cells are maximally sensitive to CDDP in G1 phase, just earlier to the beginning of DNA replication [23]. Hence, we compared the frequency distribution of cultures for at least two round of cell cycle following first cell division. In HeLa cells, treatment with CDDP alone at 48 h lead to an increase in the S-phase population at 5 & 10 μM while at 15 μM, an increase in cell death was observed (Figure 3A). However, at 72 h, low concentrations of CDDP lead to G2/M or a late S-/early G2/M block and at higher concentration, an increased cell death was observed (Figure 3C). Combining 5 μM of PJ34 with low dose (5 μM) of CDDP resulted in a significant increase in S-phase population while combining 5 μM of PJ34 with higher doses (10 μM or 15 μM) of CDDP resulted in a significant increase in cell death at 48 h (Figure 3A). At 72 h, addition of 5 μM PJ34 to 10 μM CDDP lead to increase in CDDP-mediated G2/M block, while addition of 5 μM PJ34 to 15 μM CDDP resulted in an increased cell death (Figure 3C). In HeLa cells, higher dose of PJ34 (10 μM) in combination with 5 μM CDDP resulted in an increase in S-phase population at 48 h (Figure 3B) and 72 h (Figure 3D). In contrast, combination of 10 μM of PJ34 with higher doses of CDDP (10 and 15 μM) significantly increased cell death at 48 h and 72 h (Figure 3B and 3D). Similar results were also observed in SiHa cells (Figure 3E–3H). These results indicate that combination treatment with PARP-1 inhibitor and CDDP is more effective in inducing cell death and cell cycle arrest than treatment with either of the single agents. Also, in both HeLa and SiHa, combined treatment with PARP-1 inhibitor and CDDP resulted in increase in apoptotic cells as determined by AnnexinV-PI staining, as compared to CDDP alone. However, the differences in apoptotic cells in the two groups were not statistically significant (Figure 3I and 3J).

### PARP-1 inhibition increases the anti-clonogenecity of CDDP

**Figure 4:**
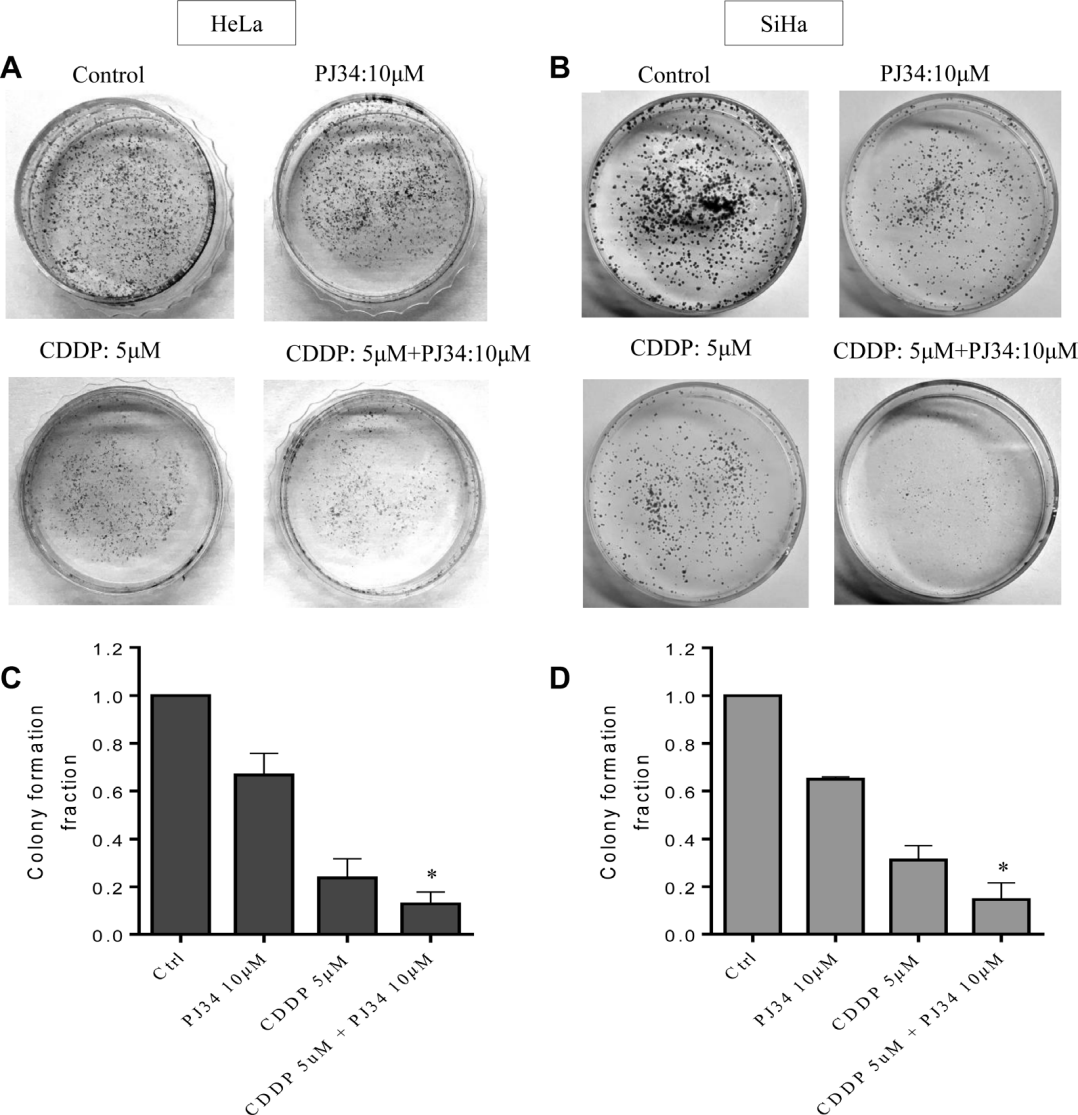
Combined effect of PJ34 & CDDP on colony formation assay. (**A–D**), Representative images for HeLa (A) and SiHa (B) cells treated with 5 μM CDDP, 10 μM PJ34 or a combination of both for 2 h. Bar graphs showing colony forming ability with respect to control of each group in HeLa (C) and SiHa (D) cells. For each doses, three replicates were performed where the survival of untreated cells (control) was set to one. Error bars represent mean ± SD (*n* ≥ 3 independent experiments). ^*^ *p < 0.05*.

Clonogenic assay is an *in vitro* cell survival assay based on competency of a single cell to create a colony. We tested colony forming ability of cervical cancer cells in presence of 5 μM CDDP alone or with 10 μM PJ34. Combined treatment with CDDP and PJ34 significantly enhanced the CDDP-mediated colony reduction of both HeLa and SiHa cells. Reduction in colony number was more pronounced in combination treatment than with either of the drug alone (Figure 4A and 4B). CDDP alone reduced the colony forming capacity of HeLa and SiHa cells to 23.66% and 31.13%, respectively, whereas combining it with PJ34 further significantly reduced the clonogenic ability to 12.75% (1.86 fold) and 15.82% (1.97 fold), respectively (Figure 4C and 4D).

### PARP-1 inhibition enhances anti-invasion/migration effect of CDDP

**Figure 5:**
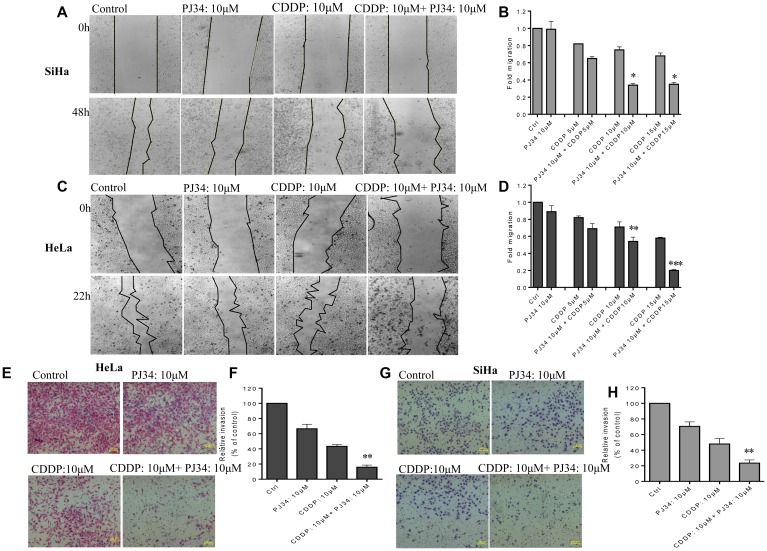
Combined effect of PJ34 & CDDP treatment on the invasion and migration. (**A–D**), representative images (under 4X magnification) of scratch wound healing assay performed after treatment of SiHa (A) and HeLa (C) cells with indicated doses of PJ34 and CDDP alone or in combination. Fold migration (with respect to control) bar graphs of each group in SiHa (B) and HeLa cells (D). (**E–H**), representative images (under 10X magnification) of invaded cells after treatment with indicated doses of PJ34 and CDDP alone or in combination in HeLa and SiHa cells. Fold change in invasion ability (with respect to control) bar graphs of each group in HeLa (F) and SiHa cells (H). Error bars represent mean ± SD (*n* ≥ 3 independent experiments). ^*^ *p < 0.05;* ^**^ *p < 0.01;* ^***^ *p < 0.001.*

We further investigated the effect of sub-lethal dose of PJ34 on CDDP-mediated inhibition of cell migration of HeLa and SiHa cells using monolayer wound-healing assay. Untreated cells exhibited a higher migration rate to the scratched wound area when compared to drug-treated cells (Figure 5). Moderate inhibition of migration was detected in both cancer cell lines treated either with PJ34 or with lower dose of CDDP, whereas a significant inhibition of migration was observed in HeLa and SiHa cells co-treated with 10 μM PJ34 and 10 or 15 μM of CDDP (Figure 5A–5D; Supplementary Figure 1).

To further study whether PARP-1 inhibition could further enhance the anti-invasion effects of CDDP in cervical cancer cells, transwell cell invasion assays was performed. Both HeLa and SiHa cells were treated with PJ34 and CDDP either alone or in combination. We observed that the number of cells invading matrigel in combinatorial treatment was greatly diminished as compared to treatment with CDDP or PJ34 alone (Figure 5). Thus PJ34 combined with CDDP significantly inhibits the invasion capacity of cervical cancer cells (Figure 5E–5H). Results obtained were further confirmed with siRNA-mediated PARP-1 suppression. Co-treatment with PARP-1 siRNA and CDDP greatly decreased the number of invaded cells as compared to CDDP alone (Supplementary Figure 2).

### PARP-1 inhibition intensifies CDDP-mediated cytotoxicity and anti-migration effect by inhibiting β-catenin signaling

**Figure 6:**
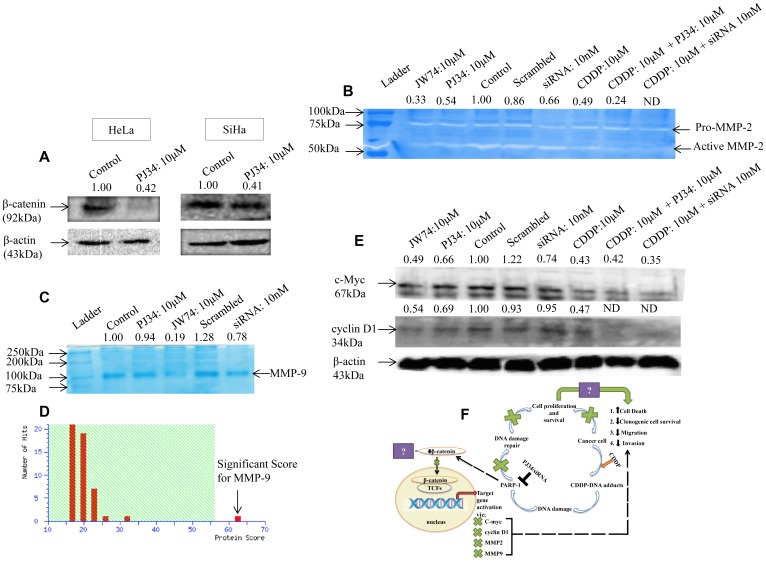
Modulation of β-catenin and its downstream signaling components by PARP-1 inhibitor alone or in combination with CDDP. (**A**) Representative immunoblots showing the expression of β-catenin upon treatment with 10 μM of PJ34 for 48 h in HeLa and for 72 h in SiHa cells. (**B**) Representative zymogram showing inhibition of MMP-2 activity in HeLa cells after treatment with PJ34 or PARP-1 siRNA as compared to control cells. Lane 2 shows inhibition of MMP-2 activity upon treatment with JW74-a specific inhibitor of β-catenin. (**C** and **D**) representative SDS-PAGE image of conditioned media proteome from HeLa cells following treatment with PJ34, PARP-1 siRNA or JW74. 50μg of protein was resolved on SDS-PAGE and stained with coomassie blue (C), followed by identification using MALDI-TOF/TOF and MS/MS analysis (D). Mascot Score Histogram: Protein score is -10*Log (P), where P is the probability that the observed match is a random event. Protein scores greater than 56 are significant (*p* < 0.05). (**E**) representative immunoblot showing expression of cyclin D1 and c-Myc in HeLa cells after treatment with PJ34 or PARP-1 siRNA as compared to control cells. β-actin was used as loading control. (**F**) model for regulation of β-catenin signaling by PARP-1 and how PARP-1 inhibition causes cell death and decreases cell metastasis, thereby, augmenting cisplatin sensitivity.

We further investigated the mechanism for anti-proliferative and anti-migratory ability of PARP-1 inhibitor. Surprisingly, PJ34 inhibited the expression of β-catenin in both the cervical cancer cell lines (Figure 6A). Hence, we hypothesized that PARP-1 inhibition might potentiates anti-migration and anti-proliferative properties of CDDP by modulating β-catenin signaling pathway. To prove this, we next determined the effect of PARP-1 inhibition on downstream signaling components of β-catenin pathway. The effect of PARP-1 inhibition on invasion potential was also evaluated through gelatin zymography. Conditioned media from HeLa cells treated with PJ34 and PARP-1 siRNA was used for zymography and cell lysate was used for immunoblotting. We found a decrease in MMP-2 activity in concentrated conditioned media derived from HeLa cells treated with PJ34 (10 μM) and 10 nM siRNA as compared to untreated cells (Figure 6B). Also, the decrease in MMP-2 activity was more significant when cells were co-treated with PJ34 and CDDP as compared to CDDP alone (Figure 6B). We used JW74, which is a known β-catenin inhibitor, as a positive control. Also, we could not find any detectable MMP-9 activity, which may be due to lower activity of MMP-9 in these cells [24]. Next, the conditioned media proteomes of HeLa treated with indicated treatment were resolved on 8% SDS-PAGE and compared with untreated control. A decrease in expression of protein around 92kDa was observed following treatment with PJ34 or PARP-1 siRNA as well as JW74 as compared to control cells (Figure 6C). This protein was excised and identified using MALDI-TOF-MS. In the MALDI-TOF analysis the Mascot Score is considered significant if the protein score is above 56. The Concise Protein Summary Report for tryptic peptides is as follows: MMP9_HUMAN MASS: 79492 SCORE: 62 EXPECT:0.011 Matches: 16 Matrix metalloproyeinase-9 OS = Homo sapiens GN = MMP9 PE = 1 SV = 3. Mass spectrometry revealed it as MMP-9 with a MASSCOT score of 62 (Figure 6D).

The effect of PJ34 mediated PARP-1 inhibition on the expression of cyclin D1 and c-Myc, which are downstream targets of β-catenin, was also checked. A noticeable decrease in the protein expression of cyclin D1 and c-Myc was observed. Decrease in the expression of both proteins upon PJ34 treatment was comparable to inhibition with JW74. Any change in expression of cyclin D1 and c-Myc was also checked with PARP-1 siRNA treatment where a significant decrease in the expression of c-Myc but not cyclin D1 was observed. Also, combination of PARP-1 inhibition and CDDP was more efficient in inhibiting the expression of both the proteins as compared to CDDP alone (Figure 6E).

## DISCUSSION

Till date, CDDP is the most effective first-line therapy for cancer cervix (4). However, the major problem with CDDP is the development of resistance during the course of treatment [8]. Escalation of CDDP dose to overcome resistance causes further cytotoxicity to dose-limiting bystander normal tissues [25]. Enhancing the efficacy of CDDP-sensitivity requires a mean to potentiate the CDDP cytotoxicity and overcome acquired resistance. In this study, we examined whether pharmacological or genetic abrogation of PARP-1 could confer enhanced CDDP sensitivity in two cervical cancer cell lines, HeLa (HPV 18 positive, adenocarcinoma) and SiHa (HPV 16 positive, squamous cell carcinoma).

Our results indicate that SiHa cells are more resistant to cisplatin than HeLa cells, which is in line with earlier findings [26, 27] this also holds true for PARP-1 inhibitor PJ34 (Figure 1B and 1C). Upon DNA damage, PARP-1 is recruited to damaged site and catalyze poly(ADP-ribosyl)-ation of DNA damage repair machinery and local substrates like histones by utilizing NAD in ATP-dependent reaction [28]. This provides negative charge to histones, creating electrostatic repulsion and causing supercoiled DNA to unwind [29]. PJ34 acts as a substrate analogue of NAD and therefore competitively inhibits PARP-1 activity [12]. FDA has approved PARP-1 inhibitors like olaparib, niraparib and rucaparib as monotherapy for BRCA-mutated breast and ovarian cancer patients [30]. Hence, we evaluated the efficacy of PJ34 to decrease cell vaibility in cervical cancer cells and compared it with CDDP. We found a time- and dose-dependent decrease in cell viability in HeLa and SiHa cells treated with PJ34 (Figure 1). PJ34 has also been found to inhibit cell growth in liver cancer cells [31] and induce cell death in melanoma cells [32]. However, we found PJ34 to be less cytotoxic than CDDP as reflected in their IC_50_ values (Figure 1). Also, we found that in combinatorial treatment, PJ34 significantly decreased the IC_50_ value of CDDP for both the cell lines and the decrease was greater than 2 folds when 10 μM PJ34 was combined with CDDP (Figure 2).

We next determined the combinatorial effects of PJ34 and CDDP on cell viability, proliferation, cell cycle progression, invasion and migration and apoptosis to evaluate if PJ34 can enhance the cytotoxicity of CDDP. For this, we chose sub-lethal doses of PJ34 5 μM & 10 μM (Figure 1) and 3 different doses of CDDP 5 μM, 10 μM and 15 μM. We first show that sub-lethal doses of PJ34 can efficiently inhibit PARP-1 activity as detected by immunoblotting using anti-PAR antibody (Figure 1H and 1I). Further, a combination of PJ34 (5 μM) and low dose of CDDP (5 μM) significantly decreased cell viability in HeLa and SiHa cells in time- and dose-dependent manner and their combined effect was synergistic (Supplementary Table 1) and superior to either of the single agent (Figure 2). Similar results were also seen in other studies where PJ34 enhanced sensitivity to CDDP in non-small cell lung cancer cells [14] and triple negative breast cancer cells [33]. Surprisingly, at higher doses of CDDP i.e. 10 μM and 15 μM, we could not find any significant differences in cell viability between CDDP alone or in combination with PJ34 (5 μM) in both HeLa and SiHa cells (Figure 2). This could be possibly due to higher cytotoxicity of CDDP itself at its higher doses.

Besides DNA repair, PARP-1 is known to interact with Ataxia Telangectasia Mutated (ATM) and ATM and Rad3-related (ATR) genes and therefore can alter the cell cycle progression and cause cell cycle arrest [34]. Inhibitors of PARP-1, either as single agent or in combination with DNA damaging agents, are known to cause G2/M mitotic arrest and/or apoptosis depending upon the extent of DNA damage [34]. PJ34 also causes a concentration dependent cell cycle arrest in cancer cell lines with distinctive genetic subtype [34]. Hence, we next analyzed the combined effect of PJ34 and CDDP on cell cycle progression using flow cytometry and PI staining. At doses chosen, PJ34 alone didn’t influence cell cycle progression in HeLa and SiHa cells, whereas low dose of CDDP (5 μM) caused late S/earlyG2-M phase block. A marked increase in cell death was observed when the dose of CDDP was increased to 10 μM and 15 μM. Further, combined treatment with PJ34 & CDDP enhanced CDDP-mediated cell cycle block/cell death when compared with CDDP alone (Figure 3). Similarly, PARP-1 inhibition enhanced the number of cells undergoing apoptosis (Figure 3) and significantly sensitized HeLa and SiHa cells to CDDP-mediated anti-clonogenic effect as compared to CDDP or PJ34 alone (Figure 4).

EMT switch and the ability to invade and metastasize is the hallmark of aggressive tumors [35, 36]. Therefore, we next tested the efficacy of PJ34 and CDDP alone or in combination in suppressing invasion and migration capabilities of HeLa and SiHa cells. Wound area covered by migrating cells upon treatment with only CDDP was higher as compared to combined treatment with PJ34 and CDDP in both the cell lines. At higher doses of both PJ34 and CDDP, wound area following treatment was determined to be almost equal to wound area at 0 h of treatment. Therefore, combination with PARP-1 inhibitor efficiently enhanced anti-migration ability of CDDP (Figure 5A–5D; Supplementary Figure 1). Likewise, numbers of invading cells were also significantly less when cells were treated with a combination of PJ34 and CDDP as compared to only CDDP or PJ34 (Figure 5E–5H). Similar results were also observed with PARP-1 siRNA in combination with CDDP (Supplementary Figure 2). These results indicate that PARP-1 inhibitor potentiates anti-metastatic activities of CDDP in cervical cancer cells and such strategy can be explored further for the management of highly metastatic tumors.

The role of PARP-1 inhibitors in decreasing DNA damage repair and promoting synthetic lethality in BRCA-mutated cancer is already well known [37]. Next, we investigated whether PJ34-mediated chemo-sensitization of cervical cancer involved any key signaling pathway. We focused on β-catenin signaling since it is known to regulate cell invasion, migration and metastasis [38–40]. Further, a functional interaction of PARP-1 with TCF-4 and β-catenin transcriptional complex is also documented [41, 42]. Interestingly, we found that PJ34 inhibited the expression of β-catenin at protein level in HeLa and SiHa cells (Figure 6A). We further explored the effect of pharmacological or genetic abrogation of PARP-1 on downstream components of β-catenin signaling. PARP-1 inhibition through PJ34 or siRNA decreased MMP-2 activity as well as the expression of MMP-9, cyclin D1 and c-Myc (Figure 6B–6E). As compared to CDDP alone, combinatorial treatment further significantly enhanced this effect. This is the first study which shows that PARP-1 inhibitor potentiates cytotoxicity of CDDP by suppressing β-catenin signaling (Figure 6F), thereby potentially inhibiting TCF-4 mediated transcription of various genes involved in cell proliferation, invasion and migration.

In conclusion, our data provides experimental evidence on the contribution of PARP-1 inhibition in enhancing the cytotoxicity of CDDP in cervical cancer cells. We also present novel findings on the suppression of, β-catenin and its downstream signaling components by PARP-1 inhibitor. We propose that PARP-1 inhibitors can be used in combination with cytotoxic chemotherapeutic agents such as CDDP to enhance the cytotoxicity of chemotherapeutic agent and to inhibit key signaling pathways involved in cell proliferation, invasion and metastasis. However, further preclinical and *in vivo* studies are warranted to validate these findings and before clinical utility of such therapeutic approach can be exactly determined.

## MATERIALS AND METHODS

### Chemicals and cell culture

Unless otherwise stated, chemicals were purchased from Sigma-Aldrich, FBS from Gibco, plastic ware from Nunc and Corning and antibodies from Santa Cruz. PJ34 (PARP-1 inhibitor), and siRNAs for PARP-1 suppression were purchased from Santa Cruz. JW74 (β-catenin inhibitor) was purchased from Sigma-Aldrich. CDDP used in study was KEMOPLAT (1mg/ml Cisplatin Injection) from Fresenius Kabi. We used HeLa and SiHa cells, both of which carry wild type BRCA1/2 (https://www.atcc.org/~/media/A6C0375544E34958B5B55C1485CB35F4.ashx), [43]. Both cell lines were cultured in DMEM high glucose supplemented with 10% FBS and 1% antibiotic-antimycotic solution and authenticated in lab using HPV16 URR type specific PCR for SiHa and HPV 18 E6 type specific PCR for HeLa on November 30, 2016. Cultures were maintained in a 37° C, 5% CO2 humidified atmosphere. Cells were seeded in appropriate plastic-ware and allowed to adapt and recover normal growth rate for overnight before drug treatment. Both cell lines were used within 30 passages after thawing and tested for mycoplasma contamination by DAPI staining/fluorescence microscopy.

### Assessment of drug dose response by cell growth

Generation time was calculated to be 22.06 h for HeLa and 40.33 h for SiHa (Figure 1A). Through MTT-based cytotoxicity assay, concentration as well as time-dependent response of PJ34 and CDDP was analyzed at 24–72 h for HeLa and 48 h–96 h for SiHa on cell survival. IC_50_ value was calculated and used to determine drug concentration for further experiments. For CDDP treatment, 5 μM, 10 μM, and 15 mM doses were chosen and for PJ34, minimum dose to efficiently inhibit PARP-1 activity were chosen i.e. 5 μM and 10 μM. Stocks for PJ34 and CDDP were prepared in normal saline and standard growth medium was used for the untreated cells.

### Combination index (CI value)

Cells were treated with constant ratio of CDDP in combination with PJ34 and CI value was evaluated using the method proposed by Chou and Talalay [44]. The CI was calculated using the formula:

$$\begin{array}{c} \text{CI}={\text{C}}_{\text{A,X}}+{\text{C}}_{\text{B,X}}\\ {\text{IC}}_{\text{X,A}}\;{\text{IC}}_{\text{X,B}} \end{array}$$

Where, C_A,X_ and C_B,X_ represents the concentration of drug A and B used in combination to attain x % drug effect. And, ICX,A and ICX,B are the concentrations for single drug required to achieve the same effect. CI value < 1 indicates synergic, CI = 1 indicates additive, and CI > 1 indicates antagonistic effect.

### PARP-1 silencing by siRNA

All transfections were performed in antibiotic-free medium. Upon reaching 60–70% confluency, cells were transiently transfected with PARP-1 siRNA in Optimem™ medium. Lipofectamine™RNAi_MAX_ reagent (Invitrogen, USA) was used and for an effective suppression of PARP-1, reverse transfection was carried out as per manufacturer’s protocol. Cells were incubated with the complex either alone or in combination with CDDP for 48 h.

### Cell vaibility assay

5 × 10^3^ cells were seeded per well in a 96-wells plate and treated with indicated doses of PJ34/CDDP alone and in combination. Each dose was replicated in 6 parallels and repeated thrice to validate results. After the treatment, medium was replaced to a fresh one containing 0.5% MTT and incubated for an additional 4 h. Resulted formazan crystals were dissolved in 100 μl DMSO. The absorbance at 570 nm was read using a multi-well spectrophotometer (Bio-Tek, USA). The concentration of the water-insoluble formazan dye was proportional to the number of living cells. Effect of respective treatment on the cell survival with respect to untreated cells was determined and also combined effect of PJ34 and CDDP treatment on CDDP IC_50_ per se was evaluated.

### Immunoblotting

Following above-mentioned treatment, the total protein was extracted using RIPA lysis buffer (ThermoScientific) supplemented with protease inhibitor cocktail (Amresco). The protein concentration was measured using BCA method (ThermoScientific). Protein (40 μg/lane for c-Myc and cyclin D1, and 60μg/lane for PARP1, PAR and β-catenin) in whole-cell lysates were resolved on 10% SDS-gel and transferred to PVDF membrane. The membranes were blocked with 5% skim milk (PARP-1, PAR and β-catenin) or 3% BSA (c-Myc, cyclin D1) in tris-buffered saline and exposed to primary antibody directed against PAR (Cat. No. Sc-56198, 1:500), PARP-1 (Cat. No. Sc-8007, 1:500), β-catenin (Cat. No Sc-7963, 1:500), c-Myc (Cat. No. Sc 764, 1:1000) and cyclin D1 (Cat. No. Sc 753 1:1500) at 4° C overnight, followed by incubation with a HRP-tagged anti-mouse/anti-rabbit secondary antibody (Cat. No. Sc-2031, 1:2000 and Cat. No. Sc-2780, 1:2000 respectively) for 2 h at room temperature. Peroxidase labeling was visualized using FluorChem E® Cell Biosciences imaging system. GAPDH or β-actin was used as housekeeping control.

### Determination of apoptosis and cell cycle progression

For cell cycle analysis, cells were seeded in six-well plates (0.5 × 10^6^ cells/well). Cells were synchronized by serum deprivation prior to experiment in incomplete culture medium overnight. Following overnight incubation, cells were treated with the PJ34/CDDP alone and in combination at indicated doses and time point. Following treatment, the cells and debris were harvested, washed with PBS and fixed in 70% ethanol at –20° C for 24 h. The cells were then incubated with PBS containing 200 μg/ml RNase A for 45 min at 37° C and 50 μg/ml propidium iodide (PI) for 15 min at room temperature in dark. Cell cycle samples were analyzed in a FACS Calibur flow cytometer (Becton Dickinson) using Becton Dickinson CellQuest software (version 5.2.1; Becton Dickinson). To determine if combination of PJ34 and CDDP induces apoptosis, fluorescein isothiocyanate (FITC) Annexin V Apoptosis Detection Kit I (BD Biosciences) was used and apoptosis was quantitatively assessed according to the manufacturer’s protocol.

### Clonogenic survival assay

Cells were seeded in a very low density (500 for untreated and 2000 for treated) in PD_35_ and allowed to adhere overnight. Following PBS wash, cells were treated with indicated doses of drug for 2 h. Drug was then washed out with 2 gentle PBS wash and cells were allowed to grow and form colony (atleast 30 cells/colony in untreated cells) for next 10 days. Resulted colonies were fixed with fixing solution (methanol:glacial acetic acid; 3:1) and stained with 0.5% crystal violet and counted under microscope.

### Cell invasion assay

The invasive ability of cells upon treatment with CDDP alone and PJ34/ PARP-1 siRNA alone or in combination with CDDP was assessed using a 24-well matrigel invasion chamber (8-μm pore size, Corning,) following manufacturers’ protocol and compared to untreated cells. Briefly, 2 × 10^4^ (HeLa) or 4 × 10^4^ cells (SiHa) cells were seeded into the transwell insert (upper chamber) in serum-free medium with/without drug. And the culture medium with 10% FBS was added in the lower chamber (the space between the well bottom and the insert) for chemo-attractant. After 24 h incubation, the insert was taken out and the cells were fixed with 4% paraformaldehyde for 30 min and stained with hematoxylin and counterstained with eosin. Non-invaded cells were carefully removed from the upper surface of the insert membrane with cotton bud. The number of migrated cells was quantified using microscope under 5 random fields from each group. Data were expressed as the relative invasion in treated cells as compared to the control cells.

### Monolayer wound healing assay

The cells were seeded in 6-wells plates at high density and grown to 90% confluence. Monolayer was scrapped parallel to create wound by using a 10 μL sterile micropipette tip, and followed by PBS wash to remove the cell debris. Cells were then incubated with fresh DMEM medium with low serum containing indicated doses of PJ34 and CDDP. The “wounded” areas were photographed by inverted microscope at 4X at 0 h and time point when approx. 50% of wound was closed in untreated cells. The relative migration distance in untreated cells was calculated by the following formula: percentage of wound closure (%) = 100 (A–B)/A, where A is the width of cell wounds at 0 h and B is the width of cell wounds after incubation. For treated cells, the inhibition effect was expressed after normalization with control.

### Determination of matrix metalloprotease activity by gelatin zymography

Gelatin zymography was performed to measure the activity of MMP according to protocol described by Sharma *et al* [45] with slight modifications. Briefly, 0.5 × 10^6^ cells were seeded in complete DMEM in a 6-wells plate and allowed to adhere overnight. Adhered cells were washed with PBS and treated with mentioned doses of PJ34/ PARP-1 siRNA)/CDDP or indicated combination in 2% serum containing culture medium. After incubation, the conditioned media was concentrated 5X through FREEZE DRAYER-5 and electrophoresed on 8% SDS-PAGE gel containing 0.5% gelatin. The gel was washed thrice with washing buffer (2.5% triton X-100, 50 mM tris-HCl, pH 7.4, 5 mM calcium chloride dehydrate, 1 μM zinc sulphate) for 1 h to remove SDS and was then incubated in reaction buffer (50 mM tris-HCl, pH 7.4, 5 mM calcium chloride dehydrate, 1 μM zinc sulphate) at 37° C for 44 h for digestion of gelatin. The gelatinolytic activity of MMP-2 was visualized by staining the gel with 0.5% Coomassie brilliant blue R 250 for overnight and destaining with destain buffer (40% methanol, 10% acetic acid, 50% water) until transparent clear band against the stained gelatin blue background appeared.

### Mass spectrophotometry

Following indicated treatment, conditioned media was electrophoresed on 8% SDS-PAGE gel and stained with coomassie blue for analysis by MALDI-TOF/TOF MS (Sandor Life Sciences Pvt. Ltd., India). Briefly, approximately 92kDa band was cut and destained using K_3_[Fe(CN)_6_] and hypochlorite, followed by dehydration using acetonitrile (ACN). After DTT treatment, sample was digested overnight using trypsin at 37° C. Resulted tryptic peptides were extracted using trifluoroacetic acid (TFA), vacuum dried and dissolved in TA buffer. The peptides obtained were mixed with HCCA (α-Cyano-4-hydroxycinnamic acid) matrix (5 mg/mL α-Cyano- 4-hydroxycinnamic acid in 1:2 ratio of 0.1% TFA and 50% ACN) in 1:1 ratio and the resulting 2μl was spotted onto the MALDI plate [(MTP 384 ground steel (Bruker Daltonics, Germany)]. After air drying the sample, it was analyzed on the MALDI TOF/TOF ULTRAFLEX III instrument (Bruker Daltonics, Germany) having smart laser beam and external calibration was done with standard peptide (PEPMIX Mixture) supplied by Bruker, with masses ranging from 1046 to 3147 Da. Further analysis was done with FLEX ANALYSIS SOFTWARE (Version 3.3) in reflectron ion mode with an average of 500 laser shots at mass detection range between 500 to 5000 m/z for obtaining the MS-MS. The masses obtained in the MSMS were submitted for Mascot search in “CONCERNED” database for identification of the protein.

### Statistical evaluation

Data were expressed as the mean ± standard deviation from at least three independent experiments. Comparisons between groups were performed using the two-tailed Student’s *t* test. Analysis was done using GraphPad Prism-6. Results were considered to be statistically significant when *p* < 0.05.

## SUPPLEMENTARY MATERIALS



## Abbreviations

CTRL: Control
PARP-1: Poly (ADP-ribosyl) polymerase-1
PAR: Poly(ADP-ribose) Polymer
CDDP: Cisplatin
PJ34: N-(6-Oxo-5,6-dihydrophenanthridin-2-yl)-(N,N-dimethylamino)acetamide hydrochloride
MTT: 3-4,5-demethylthylthiazol-2yl] -2,5 diphenyltetrazoliumbroide
PARP-1i: Poly (ADP-ribosyl) polymerase-1 inhibitor
PARP-1 siRNA: siRNA against PARP-1
FBS: Fetal Bovine Serum
