# Correction: PARP-1 inhibitor modulate β-catenin signaling to enhance cisplatin sensitivity in cancer cervix

**DOI:** 10.18632/oncotarget.27101

**Published:** 2019-07-30

**Authors:** Minakshi Mann, Sachin Kumar, Shyam S. Chauhan, Neerja Bhatla, Sunesh Kumar, Sameer Bakhshi, Ritu Gupta, Ashok Sharma, Lalit Kumar

**Affiliations:** ^1^ Department of Medical Oncology, Dr. B.R.A. Institute Rotary Cancer Hospital, All India Institute of Medical Sciences, New Delhi, India; ^2^ Department of Biochemistry, All India Institute of Medical Sciences, New Delhi, India; ^3^ Department of Obstetrics and Gynecology, All India Institute of Medical Sciences, New Delhi, India; ^4^ Laboratory Oncology Unit, Dr. B.R.A. Institute Rotary Cancer Hospital, All India Institute of Medical Sciences, New Delhi, India

**This article has been corrected:** Dr. Ashok Sharma has been named as co-corresponding author on this paper, and has been moved from third place to second-to-last author in the listing.

The other correction, regarding funding, has already been made.

 Original article: Oncotarget. 2019; 10:4262–4275. 4262-4275 . https://doi.org/10.18632/oncotarget.27008

