# Supplementary material for: PARP-1 inhibitor modulate β-catenin signaling to enhance cisplatin sensitivity in cancer cervix
Source: Oncotarget. 2019 Jul 2;10(42):4262–75. doi: 10.18632/oncotarget.27008 (PMC6611509; doi:10.18632/oncotarget.27008)
Supplement: Supplementary file 1 [file oncotarget-10-4262-s001.pdf]

PARP-1 inhibitor modulate β-catenin signaling to enhance cisplatin sensitivity in cancer cervix

SUPPLEMENTARY MATERIALS

Supplementary Table 1: CI* values for combined treatment of CDDP and PJ34

**Drug combination[#]**<br>**Time points**	**CDDP: 5 µM +**<br>**PJ34: 5 µM**	**CDDP: 5 µM +**<br>**PJ34: 10 µM**	**CDDP: 10 µM +**<br>**PJ34: 10 µM**
48h (HeLa)	---	0.97	---
72h (HeLa)	0.94	0.91	---
48h (SiHa)	0.64	0.51	0.77
72h (SiHa)	0.83	0.67	0.92

[*]CI value < 1 indicates synergic, CI = 1 indicates additive, and CI > 1 indicates antagonistic effect. [#]CI values has been calculated for combinatorial treatment of CDDP and PJ34 leading to significant decrease in cell vaibility as compared to either of the single agent alone.

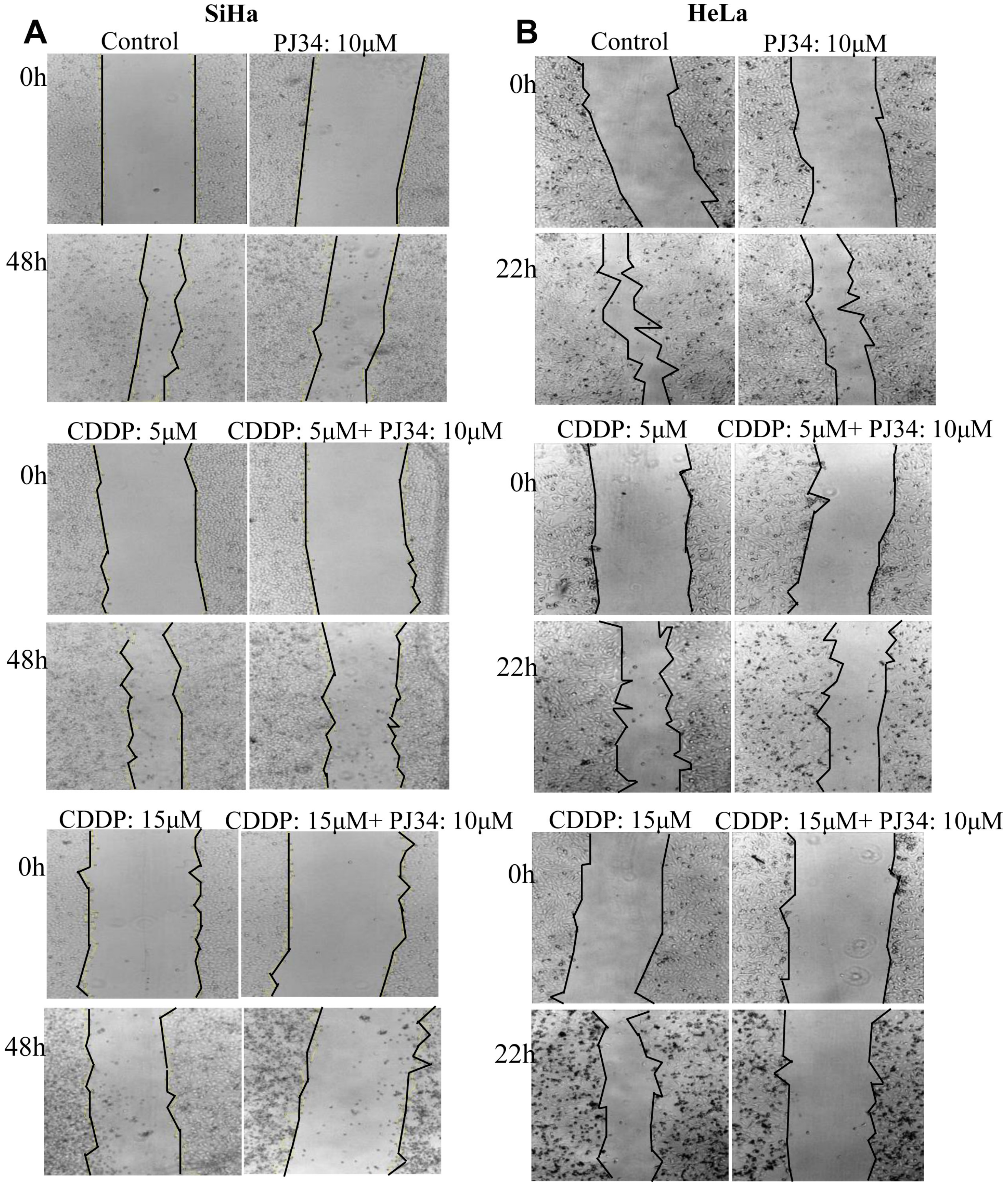

Supplementary Figure 1: Combined effect of PJ34 & CDDP treatment on the cell migration. Representative images (4X magnification) of scratch wound healing assay performed after treatment of SiHa (**A**) and HeLa (**B**) cells with indicated doses of PJ34 and CDDP alone or in combination.

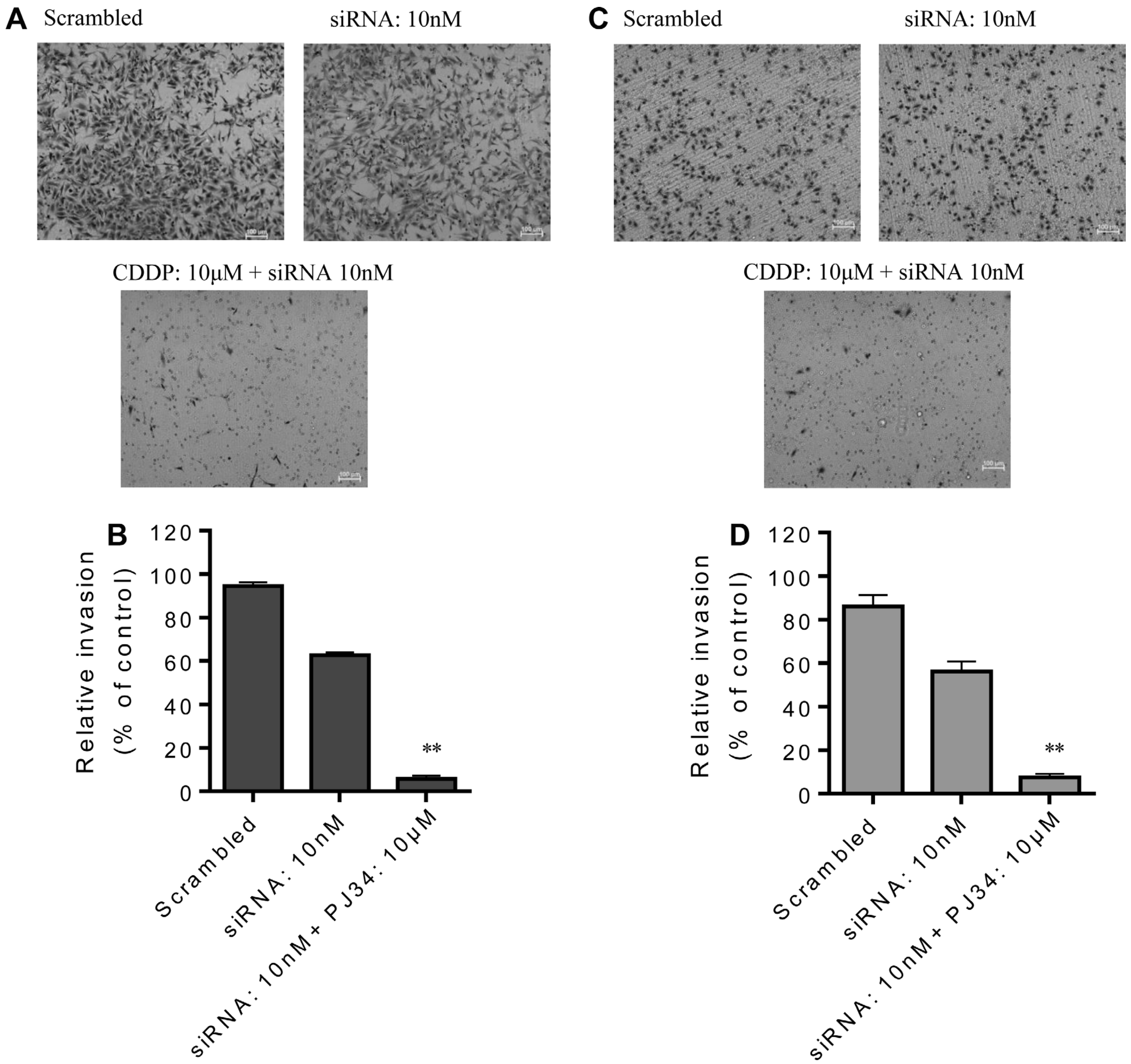

Supplementary Figure 2: Combined effect of PARP-1 siRNA & CDDP treatment on the cell invasion. Representative images (under 10X magnification) of invaded cells after treatment with indicated doses in HeLa (**A**) and SiHa (**C**). Fold change in invasion ability (with respect to control) bar graphs of each group in HeLa (**B**) and SiHa cells (**D**). Each value represents a mean value ± SD of at least three independent experiments. $^{**}p < 0.01$.
